# One-year restoration of vaginal health: synergistic dynamics of microbiome and metabolome following the elimination of high-grade cervical intraepithelial neoplasia

**DOI:** 10.1128/msystems.01190-25

**Published:** 2025-11-17

**Authors:** Hui Du, Xin Jiang, Yu Liu, Jun Hou, Changzhong Li, Ruifang Wu, Shuai Cheng Li, Wenkui Dai

**Affiliations:** 1 Department of Obstetrics and Gynecology, Peking University Shenzhen Hospital 74573, Shenzhen, China; 2 Institute of Obstetrics and Gynecology, Shenzhen PKU-HKUST Medical Center 610537 , Shenzhen, China; 3 Shenzhen Key Laboratory on Technology for Early Diagnosis of Major Gynecologic Diseases, Shenzhen, China; 4 Department of Computer Science, City University of Hong Kong 622765 , Hong Kong, China; Child Health Research Foundation, Dhaka, Bangladesh

**Keywords:** cervical intraepithelial neoplasia, therapeutic elimination, vaginal microbiota, metabolomics, human papillomavirus

## Abstract

Therapeutic elimination of high-grade cervical intraepithelial neoplasia (CIN) is widely implemented for cervical cancer prevention. Despite the demonstrated dysbiosis of vaginal microenvironment in high-grade CIN, its post-therapy restorations remain to be poorly understood, especially in functional aspects. This study aimed to characterize temporal changes in both vaginal microbiota (VM) and metabolome (VMeta) following therapeutic elimination of high-grade CIN. We conducted a longitudinal study of 32 HPV-positive women with high-grade CIN who underwent therapeutic procedures. Vaginal swabs were collected at baseline (pre-therapy) and at 6- and 12-month follow-up visits for integrated VM and VMeta analysis. We observed a gradual restoration of *Lactobacillus crispatus* levels from baseline to 12 months (*P* < 0.05). Concurrently, we detected significant decreases in dysbiosis-associated bacteria, including *Prevotella bivia*, *Ureaplasma parvum*, and *Peptoniphilus* sp. 6 months post-therapy compared to the baseline. VMeta analysis revealed distinct metabolic shifts across the follow-up periods. The early post-therapy phase (baseline to 6 months) was characterized by enrichment of glycerophospholipids and depletion of nucleotide metabolites, while the later phase (6–12 months) showed increases in flavonoids, lysophospholipids, bioactive amides, and amino acid metabolism. Integration of correlation and dynamic Bayesian network analysis indicated potential regulatory relationships and time-lag effects involving HPV infection, *L. crispatus*, *Bifidobacterium* sp., *Streptococcus anginosus*, *Megasphaera* sp., *U. parvum,* and those metabolites. This study enhances our understanding of a sequential restoration process post-therapy in the vaginal microenvironment.

**IMPORTANCE:**

Therapeutic elimination of high-grade CIN is routine, yet functional recovery of the vaginal ecosystem is poorly defined. In a 12-month longitudinal multi-omics study of 32 women, we show stepwise restoration: progressive *L. crispatus* dominance with sustained decreases in dysbiosis-associated taxa (*P. bivia, U. parvum, Peptoniphilus*). Metabolically, an early rise in glycerophospholipids and fall in nucleotide metabolites is followed by later enrichment of flavonoids, lysophospholipids, bioactive amides, and amino acid derivatives. Correlation and dynamic Bayesian network analyses reveal putative regulatory links, time-lag effects, and downstream impacts of HPV clearance. These findings deliver a functional roadmap of post-therapy recovery, nominate measurable microbial–metabolite milestones and candidate biomarkers for monitoring, and suggest targets for adjunct interventions to accelerate re-establishment of protective states. This work informs precision follow-up in cervical cancer prevention programs.

## INTRODUCTION

Therapeutic elimination procedures are widely implemented for high-grade cervical intraepithelial neoplasia (CIN) (1, 2), a condition characterized by dysbiosis of the vaginal microbiota (VM) (3–7). Growing evidence indicates that HPV infection modulates host responses that affect VM compositions (8, 9). In consistency, increased *Lactobacillus* abundance has been observed following CIN elimination (10–12). This suggests the restoration of vaginal health via the re-establishment of *Lactobacillus* dominance post-therapy.

Recent metabolomic investigations have revealed distinct vaginal metabolome (VMeta) profiles in women with high-grade CIN compared to HPV-negative women without cervical lesions (3, 13–15). Ilhan et al. demonstrated an enrichment of lipid metabolites with concurrent depletion of amino acid and nucleotide metabolites in women with cervical dysplasia, linking these metabolic alterations to vaginal dysbiosis and HPV infection (3). Interestingly, anti-inflammatory nucleotide levels correlated with *Lactobacillus* dominance. Other researchers have documented increased concentrations of biogenic amines and phospholipids in HPV-positive women compared to their HPV-negative counterparts (13).

Emerging research demonstrates that dynamic changes in structural compositions differ significantly from those in functional compositions, with the latter playing crucial roles in VM-host interactions (13–15). Recent findings indicate that metabolomic profiling offers superior performance in assessing associations between vaginal microenvironment, HPV infection, and CIN progression (10, 15). These observations underscore the importance of investigating post-therapy dynamics of functional communities within the vaginal microenvironment.

Besides traditional LEEP surgery, several therapeutic approaches are employed for high-grade CIN, including CO₂ cryotherapy and thermal ablation (16–18). CO₂ cryotherapy involves the application of extremely cold carbon dioxide to destroy abnormal cervical tissue, while thermal ablation utilizes heat to achieve similar results, either with single or multiple probes. The choice between these techniques depends on various factors, including lesion size, location, clinician expertise, and equipment availability. These treatments are all effective in removing abnormal cervical tissue while preserving cervical function. However, there remains a significant gap in population-based studies examining the post-therapy synergistic dynamics of VM and VMeta, particularly in developing countries where therapeutic elimination of precancerous CIN is a cornerstone of cervical cancer prevention. To address this knowledge gap, we conducted a 12-month longitudinal follow-up study after initial therapies. Following stringent inclusion criteria, 32 women were enrolled for integrated VM and VMeta analysis. Our objective was to characterize the synergistic dynamic changes between VM and VMeta following therapeutic elimination of CIN and HPV infection.

## MATERIALS AND METHODS

### Study design and sample collection

HPV testing, CIN diagnosis, and sample collection followed protocols described in our previous publication (10). Briefly, we recruited HPV-positive women who underwent therapeutic procedures for high-grade CIN according to the following eligibility criteria: (i) age ≥18 years and sexually active; (ii) pre-menopausal status; (iii) no history of cervical ablation/resection, hysterectomy, or pelvic radiotherapy; (iv) absence of autoimmune diseases or HIV infection; (v) no exposure to immunosuppressants, oral antibiotics, or vaginal douching within the past month; (vi) no sexual activity within the past week; and (vii) negative results for H₂O₂, leukocyte esterase, neuraminidase, β-glucuronidase, and acetylaminoglucosidase (detected using the bPR2014A platform, Jiangsu Bioperfectus Technologies Co., Ltd.).

Vaginal swabs were collected 3–7 days after menstruation for VM and VMeta analysis before therapy and at 6 and 12 months post-therapy. From the initial cohort, 32 women met our stringent inclusion criteria: (i) availability of qualified samples at baseline and follow-up visits; (ii) HPV-negative status at both 6M and 12M visits; and (iii) negative results for all tested vaginal enzymes at follow-up visits.

### Data generation for VM and vaginal metabolome

For VM analysis, we extracted microbial DNA using the Dneasy PowerSoil Pro Kit (Qiagen, Germany) according to our previously published protocol. We amplified the V4-V5 hypervariable regions of the 16S rRNA gene using primers 515-FR (GTGCCAGCMGCCGCGGTAA) and 926-RR (CCGTCAATTCMTTTRAGTTT). PCR product quality was determined using Qubit (Thermo Fisher Scientific, Singapore). DNA libraries were sequenced with 250 bp read length on the Illumina NovaSeq platform (Illumina, San Diego, CA, USA). Sequencing data were processed through QIIME 2 to generate VM profiling tables for downstream analysis. Specifically, reads were removed when they contained more than 10 low-quality (<Q20) bases or 15 bases of adapter sequences. The filtered reads were then connected into tags (at least 50 overlapping bases based on at least 50 overlapping bases), which were clustered into operational taxonomic units (OTUs) with 97% similarity and then assigned taxonomic units.

For untargeted metabolomics analysis, swabs preserved at −80°C underwent metabolite extraction following a standardized protocol. Samples were thawed at 4°C and combined with 700 µL extraction solvent (methanol:acetonitrile:water = 4:2:1, vol/vol/vol) containing internal standards. Following vortexing and storage at −20°C for 2 h, the mixture was centrifuged (25,000 *g*, 4°C, 15 min). The resulting supernatant was dried, reconstituted in 180 µL methanol:water (1:1, vol/vol), and centrifuged again under identical conditions. The final supernatant was transferred to a new EP tube for analysis using a Waters UPLC I-Class Plus system coupled with a QTRAP 6500 Plus mass spectrometer.

LC-MS/MS data collection enabled peak extraction and identification. Metabolites were identified using Compound Discoverer 3.3 with reference to in-house metabolome database, mzCloud, and ChemSpider. The output files were processed in MetaX, which included: (i) data normalization via Probabilistic Quotient Normalization to obtain relative peak areas; (ii) exclusion of metabolites absent in >50% of quality control samples or >80% of test samples; (iii) removal of metabolites with Coefficient of Variation >30% in QC samples.

Functional annotation of metabolites was performed through comprehensive searches of the Human Metabolome Database (HMDB, version 5.0) and Kyoto Encyclopedia of Genes and Genomes (KEGG, version 96.0). The resulting metabolite abundance matrix was used for subsequent analysis.

### Bioinformatics analysis

To identify VM community state types (CSTs), we applied unsupervised hierarchical clustering based on average Euclidean distance metrics. The CST of each sample was determined according to the dominant bacterial genera or species with relative abundance ≥50%. For samples with *Lactobacillus* dominance, CST_MixedL was assigned when the total relative abundance of two *Lactobacillus* species exceeded 50%, with each individual species <50%.

Permutational Multivariate Analysis of Variance (PERMANOVA) was employed to assess the explained variance of phenotypical factors on baseline VM and VMeta compositions using the adonis2 function in the vegan package (v.2.6-10). For dynamic changes in microbial and metabolic profiles, we calculated vectors of person-specific changes for each OTU/metabolite per individual as log₂(6M_abundance/Baseline_abundance) and log₂(12M_abundance/6M_abundance). These vectors were used to quantify the explained variance of multiple factors for both VM and VMeta dynamics. Bray-Curtis distances were calculated to assess dissimilarities between samples using the vegdist function in the vegan package.

Deterministic change of each species/metabolite was assessed by consistent change coefficient >1. For each feature (species/metabolite), we computed a robust coefficient of variance (CV) ratio by comparing variability between timepoints, with Median Absolute Deviation (MAD) replacing SD to improve outlier resistance. To quantify directional uniformity of longitudinal changes, the absolute mean change (follow-up minus baseline) was divided by the MAD of changes across subjects.

Fold changes for each species and metabolite were calculated using median values. Correlations between VM and VMeta across the three timepoints were analyzed using the rmcorr package (v.0.7.0).

### Statistical analysis

We employed Wilcoxon signed-rank tests to analyze differences between Baseline and 6M, as well as between 6M and 12M groups. Significant variations in VM components were determined at *P* < 0.05. For metabolites, statistical significance was defined as *P* < 0.05 and |log₂FC| ≥ 0.585. We did not apply multiple testing correction due to the exploratory nature of this study and the relatively small sample size, which would have increased the risk of Type II errors and thus missed biologically meaningful metabolites.

Significant correlations between VM and VMeta were defined as *P* < 0.05 with 95% confidence intervals not crossing zero. All results were visualized using R software (v.4.0.5).

### Dynamic Bayesian network analysis

For dynamic Bayesian network analysis using the dbnR package (v.0.7.9), we included 62 features in the final model: HPV infection status, 12 bacterial species, and 49 metabolites that showed significant differences in either the 6M-vs-Baseline or 12M-vs-6M comparisons. To avoid local maxima, we bootstrapped 1,000 networks across 1,000 random restarts. Network structures were learned using the dmmhc method. Edges and nodes were extracted from the generated 1,000,000 networks, and edge strength was calculated using the custom.strength function in the bnlearn package (v.5.0.2). Network visualization was performed using the igraph package (v.2.1.4).

## RESULTS

### Study design

**Fig 1:**
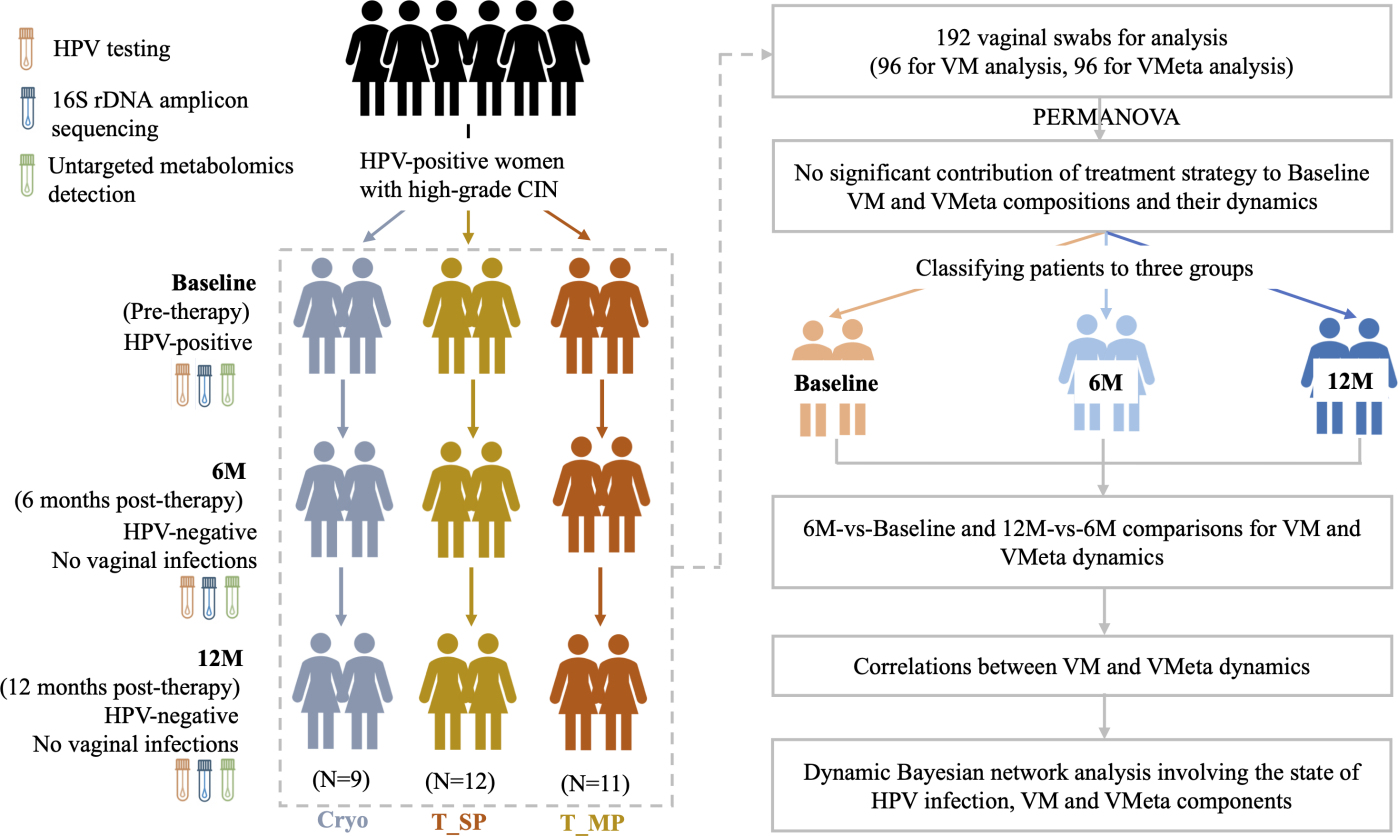
Workflow of this study. A total of 32 HPV-positive women accompanied by high-grade cervical intraepithelial neoplasia (CIN) were included. There were no HPV positivity and vaginal infections following therapeutic elimination of high-grade CIN. Synergistic dynamics of vaginal microbiota and metabolome were assessed in reaction to therapies. Regulation network were also inferred for co-altered microbiota and metabolome components.

Thirty-two HPV-positive women with high-grade CIN participated in this longitudinal study (Fig. 1, Table 1). From each participant (*n* = 32), we collected two vaginal swabs at each of the three timepoints (Baseline, 6M, and 12M): one swab for VM analysis and another for VMeta analysis. This resulted in a total of 192 samples (96 for VM and 96 for VMeta).

**TABLE 1:** Information of included 32 patients^*a*^

Therapy strategy	CO_2_ cryotherapy (Cryo)	Thermal ablation (single probe, T_SP)	Thermal ablation (multiple probes, T_MP)	*P* value
Number	9	12	11	
Age	29 (22–40)	31.9 (19–37)	29.3 (23–38)	0.6301
HPV infection				
HPV16(positive/total)	4/9	4/12	8/11	0.3258
HPV18(positive/total)	1/9	1/12	1/11	0.8297
Other 12 HPV genotypes (positive/total)	7/9	9/12	4/11	0.4729
Prophylactic vaccination				
4-valent (vaccinated/total)	1/9	4/12	1/11	0.8139
9-valent (vaccinated/total)	1/9	2/12	1/11	0.9128
Smoking	1/9	1/12	3/11	0.8403
No. of sex partner	2.7 (1–4)	3.2 (1–10)	2.9 (1–10)	0.5781
Age with first sex activity	20.4 (18–26)	21.5 (16–30)	20.8 (18–25)	0.7916

^*a*^ For age, no. of sex partner and age with first sex activity, statistic significance was assessed by Friedman test. For other factors, statistic significance was assessed via Bowker test.

Among those participants, 9 received CO₂ cryotherapy (Cryo), while 12 and 11 underwent thermal ablation with single probe (T_SP) and multiple probes (T_MP), respectively. No significant demographic or clinical differences were observed between treatment groups regarding age, HPV genotypes, and HPV vaccination history (Table 1).

Further analysis revealed no significant differences in VM and VMeta baseline profiles or post-treatment dynamics between those three therapeutic approaches (Fig. S1 and Table S1). Therefore, samples were categorized into Baseline, 6M, and 12M groups for subsequent analysis (Fig. 1).

### Dynamic changes of VM compositions 6 and 12 months after therapeutic elimination of high-grade CIN

**Fig 2:**
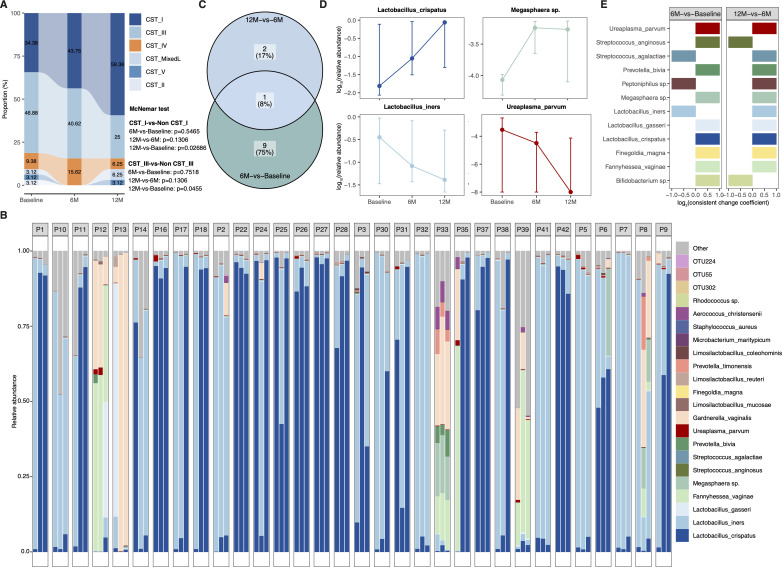
Vaginal microbiota dynamics following therapeutic interventions. (**A**) Distribution of community state type (CST) at each timepoint. CST_I: *Lactobacillus crispatus*-dominated; CST_III: *L. iners*-dominated; CST_II: *L. gasseri*-dominated; CST_V: *L. jensenii*-dominated; CST_MixedL: dominated by two *Lactobacillus* species with each having < 50% relative abundance; CST_IV: non-*Lactobacillus*-dominated. (**B**) Species-level compositions for 96 microbial samples. (**C**) Shared and distinct list of species with significant variations between 6M-vs-Baseline and 12M-vs-6M comparisons. (**D**) Dynamics of four species at three timepoints. The point represents median value. Top and bottom error bars represent upper and lower quartile, respectively. (**E**) Consistent change coefficients for 12 species with significant changes following therapies. Deterministic change was determined by consistent change coefficient > 1.

We identified 50 operational taxonomic units (OTUs) from sequenced data, annotated as 30 genera and 40 species. Although statistically non-significant (*P* > 0.05), 6M-vs-Baseline dissimilarities measured by Bray-Curtis distance were higher than those in 12M-vs-6M comparisons (Fig. S2). The proportion of *Lactobacillus crispatus*-dominated community state type I (CST_I) samples increased following therapies, with no statistical significance observed for either 6M-vs-Baseline or 12M-vs-6M comparisons (Fig. 2A). However, CST_I proportion was significantly higher at the 12M follow-up compared to Baseline (*P* < 0.05), indicating gradual restoration of *L. crispatus* abundance after therapies. Further analysis revealed that CST_I primarily evolved from *L. iners*-dominated CST_III (Fig. 2A and B).

At the threshold of *P* < 0.05, we identified 10 and 3 VM components that differed significantly in 6M-vs-Baseline and 12M-vs-6M comparisons, respectively (Fig. 2C). Among the 10 components that changed at 6M, five showed increased abundance, including *L. gasseri*, *Bifidobacterium* sp., *Streptococcus anginosus*, *Megasphaera* sp., and *Fannyhessea vaginae* (Fig. 2D; Fig. S3). Conversely, five components decreased at 6M, including *Prevotella bivia*, *L. iners*, *Ureaplasma parvum*, *Finegoldia magna*, and *Peptoniphilus* sp., with *U. parvum* continuing to decrease between 6M and 12M (Fig. 2D; Fig. S3). The remaining two VM components that differed between 12M and 6 M samples included increased *L. crispatus* and *S. agalactiae* at the 12M follow-up. Based on a consistent change coefficient >1, further analysis confirmed deterministic changes in these 12 VM components across the follow-up period (Fig. 2E).

### Vaginal metabolome variations 6 and 12 months after therapeutic elimination of high-grade CIN

**Fig 3:**
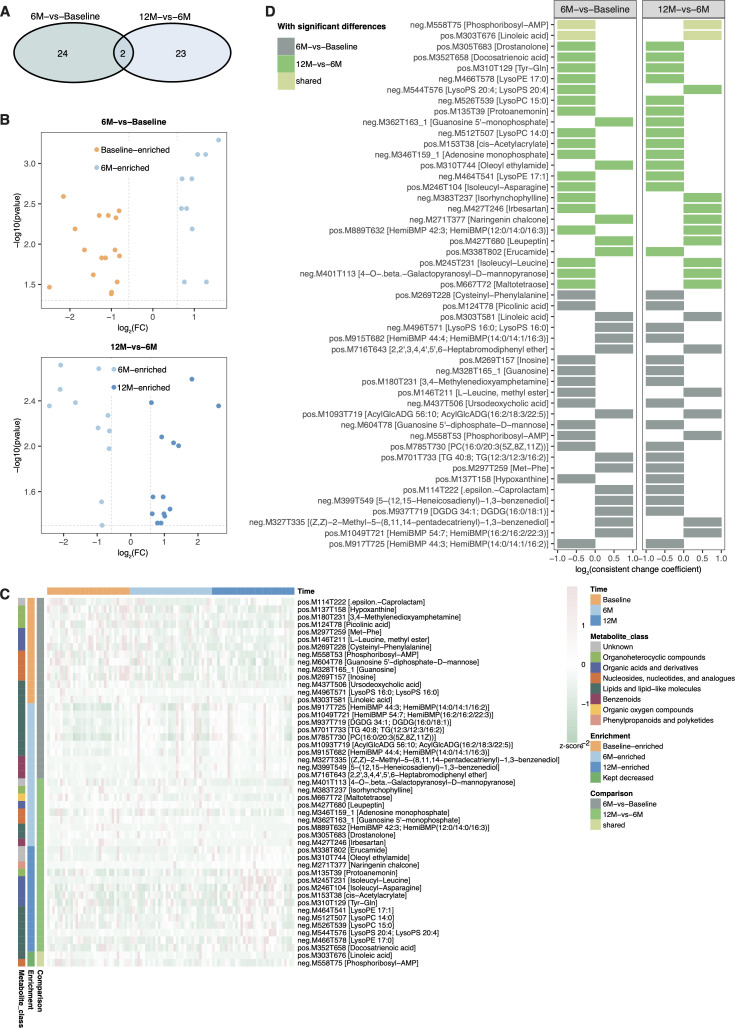
Vaginal metabolome dynamics following therapeutic interventions. (**A**) Shared and distinct list of metabolites with significant variations between 6M-vs-Baseline and 12M-vs-6M comparisons. (**B**) This plot displays 26 and 25 metabolites with significant variations in 6M-vs-Baseline and 12M-vs-6M comparisons, respectively. The statistical significance was determined by *P* < 0.05 and |log_2_FC| ≥ 0.585. (**C**) Heatmap of normalized abundance (*z*-score) of 49 metabolites. This heatmap visually represents the normalized abundance of metabolites that differentiate between 6M and Baseline or between 12M and 6M follow-up visits. The intensity of color indicates the relative abundance of each metabolite. (**D**) Consistent change coefficients for 49 metabolites with significant changes following therapies. Deterministic change was determined by consistent change coefficient > 1.

Untargeted metabolomics identified 389 metabolites with known identity at the MS2 level. Bray-Curtis distance analysis revealed no significant differences in VMeta dynamics between 6M-vs-Baseline and 12M-vs-6M comparisons (Fig. S4). However, we observed notable differences in specific metabolites between these timepoints (Fig. 3A). Using criteria of *P* < 0.05 and |log₂FC| ≥ 0.585, we identified 49 differentially produced metabolites across the 6M-vs-Baseline and 12M-vs-6M comparisons (Fig. 3A and B). Only two metabolites were common to both comparisons (Fig. 3A and C): linoleic acid (pos.M303T676) and phosphoribosyl-AMP, both of which progressively decreased from Baseline through 6M to 12M. The remaining metabolites were unique to either the 6M-vs-Baseline or 12M-vs-6M comparison.

Twenty-six metabolites showed differential levels between 6M and Baseline samples (Fig. 3B). Of these, 10 were enriched at 6M, with 8 having consistent change coefficient >1, including HemiBMP 54:7; HemiBMP(16:2/16:2/22:3), TG 40:8; TG(12:3/12:3/16:2), HemiBMP 44:4; HemiBMP(14:0/14:1/16:3), and multiple DGDG 34:1; DGDG(16:0/18:1) variants (Fig. 3C and D). Conversely, 16 metabolites were depleted at 6M, including hypoxanthine, ursodeoxycholic acid, linoleic acid, picolinic acid, and guanosine 5′-diphosphate-D-mannose. However, only 4 of these 16 metabolites had consistent change coefficient >1, namely linoleic acid(pos.M303T581), Met-Phe, ε-Caprolactam, and LysoPS 16:0.

In the 12M-vs-6M comparison, 25 metabolites showed distinct levels (Fig. 3B). Among these, 14 increased at 12M with 5 having consistent change coefficient >1, including erucamide, naringenin chalcone, oleoyl ethylamide, and LysoPS 20:4 (Fig. 3C and D). Other 12M-enriched metabolites included docosatrienoic acid, protoanemonin, cis-acetylacrylate, and isoleucyl-leucine. In contrast, 11 metabolites decreased at 12M, including maltotetraose, linoleic acid(pos.M303T676), guanosine 5′-monophosphate, adenosine monophosphate, leupeptin, 4-O-β-galactopyranosyl-D-mannopyranose, and isorhynchophylline, all with consistent change coefficient >1.

### Correlations between VM and VMeta dynamics

**Fig 4:**
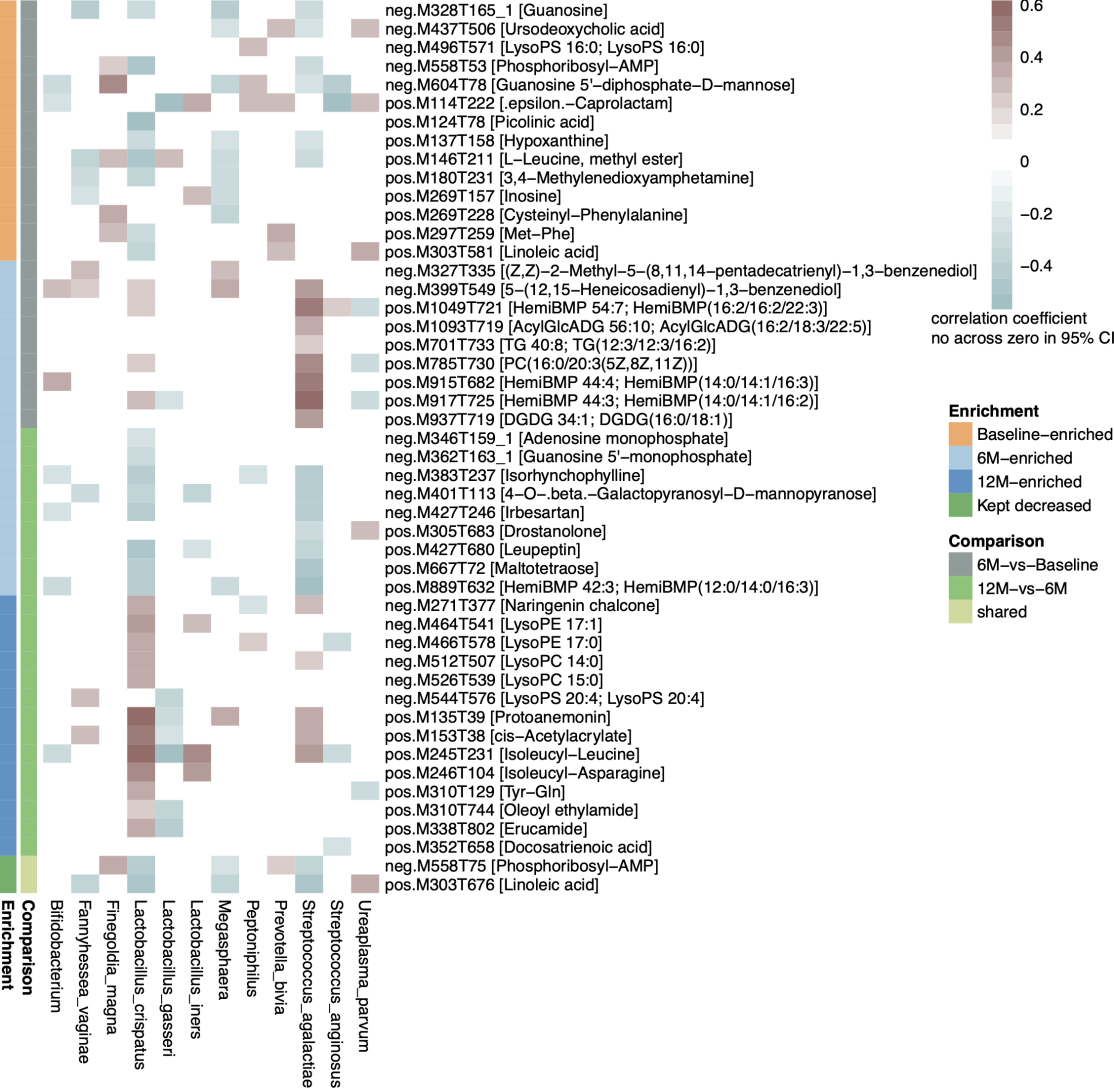
Correlations between vaginal microbiota and metabolome dynamics. This heatmap displays correlations between 12 species and 49 metabolites that varied following therapeutic interventions. Purple color represents positive correlations, while blue color represents negative correlations. The statistical significance was determined by *P* < 0.05.

Repeated measures correlation analysis demonstrated significant correlations between VM and VMeta dynamics (Fig. 4). Using criteria of *P* < 0.05 and 95% CI not crossing zero, we identified 142 significant correlations between 12 species and 48 metabolites (Fig. 4). Specifically, *L. crispatus* positively correlated with 16 metabolites, of which 4 were enriched at 6M compared to Baseline, including 5-(12,15-Heneicosadienyl)−1,3-benzenediol, HemiBMP 54:7; HemiBMP(16:2/16:2/22:3), PC(16:0/20:3(5Z,8Z,11Z)), and HemiBMP 44:3; HemiBMP(14:0/14:1/16:2). The remaining 12 metabolites were significantly enriched at 12M compared to 6M, including naringenin chalcone, various lysophospholipids (LysoPE 17:1, LysoPE 17:0, LysoPC 14:0, LysoPC 15:0), protoanemonin, cis-acetylacrylate, and several dipeptides and amides.

Conversely, *L. crispatus* negatively correlated with 18 metabolites, of which 10 were significantly depleted at 6M compared to Baseline (Fig. 4), including guanosine, phosphoribosyl-AMP, picolinic acid, hypoxanthine, L-leucine methyl ester, 3,4-methylenedioxyamphetamine, Met-Phe, and linoleic acid. Among these 18 metabolites, 9 were notably depleted at 12M compared to 6M, including adenosine monophosphate, guanosine 5′-monophosphate, isorhynchophylline, 4-O-β-galactopyranosyl-D-mannopyranose, irbesartan, phosphoribosyl-AMP, linoleic acid, leupeptin, and HemiBMP 42:3; HemiBMP(12:0/14:0/16:3).

*L. gasseri* negatively correlated with 8 metabolites (Fig. 4), including HemiBMP 44:3; HemiBMP(14:0/14:1/16:2), erucamide, oleoyl ethylamide, cis-acetylacrylate, protoanemonin, LysoPS 20:4, 4-O-β-galactopyranosyl-D-mannopyranose, leupeptin, ε-caprolactam, and isoleucyl-leucine, of which ε-Caprolactam and isoleucyl-leucine positively correlated with *L. iners*. Other metabolites positively correlated with *L. iners* included inosine, isoleucyl-asparagine, and LysoPE 17:1. In contrast, 4-O-β-galactopyranosyl-D-mannopyranose and leupeptin showed negative correlations with *L. iners*.

For non-*Lactobacillus* VM components, we identified positive correlations between 10 metabolites and 5 species that increased during follow-up (Fig. 4). Specifically, 5-(12,15-Heneicosadienyl)−1,3-benzenediol positively correlated with *Megasphaera* sp., *Bifidobacterium* sp., *S. agalactiae*, and *F. vaginae*. HemiBMP 44:4; HemiBMP(14:0/14:1/16:3) positively correlated with both *Bifidobacterium* sp. and *S. agalactiae,* while cis-acetylacrylate positively correlated with *F. vaginae* and *S. agalactiae*. Other metabolites showing positive correlations with these species included naringenin chalcone, HemiBMP 44:3; HemiBMP(14:0/14:1/16:2), and PC(16:0/20:3(5Z,8Z,11Z)), which negatively correlated with the declining *U. parvum* and *Peptoniphilus* sp.

Further analysis revealed 20 paired positive correlations between 13 metabolites and *P. bivia*, *F. magna*, *Peptoniphilus* sp., and *U. parvum*, all of which decreased significantly after therapy (Fig. 4). Specifically, ε-caprolactam, ursodeoxycholic acid, phosphoribosyl-AMP, linoleic acid, and guanosine 5′-diphosphate-D-mannose correlated with multiple declining species. These metabolites negatively correlated with multiple species that were enriched post-therapy.

### Inferred regulatory network in co-altered VM and VMeta dynamics

**Fig 5:**
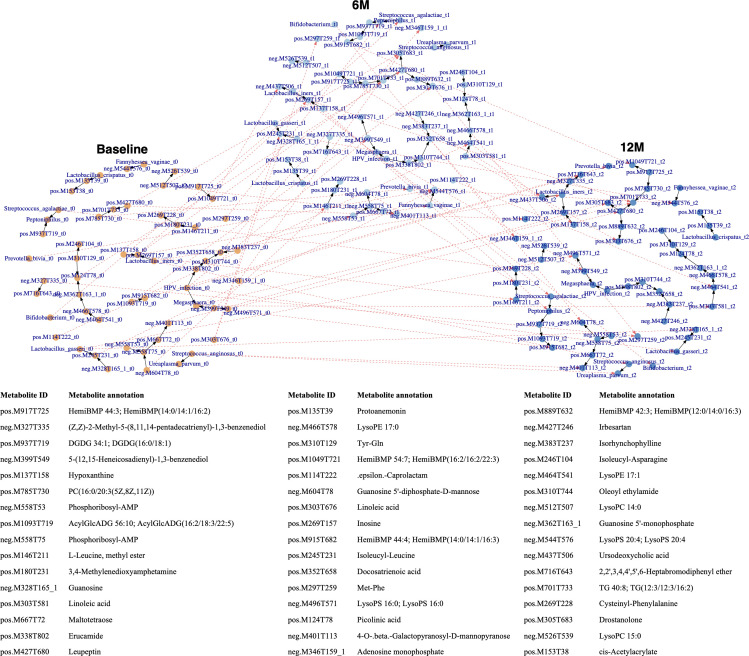
Regulatory networks inferred by dynamic Bayesian network analysis. A total of 174 edges were identified. Analysis suggested the complexity of dynamics and associated interactions among HPV infection, microbiota, and metabolome following therapeutic interventions. Black lines represent regulation network at each timepoint, while red lines represent time-lag effects.

Using dynamic Bayesian network analysis with an edge strength threshold ≥0.3, we generated 174 edges representing potential interactions between HPV infection status, VM, and VMeta dynamics (Fig. 5). The analysis revealed that increased *Bifidobacterium* sp. at 6M potentially influenced the abundance of depleted guanosine 5′-diphosphate-D-mannose at 12M. Decreased *P. bivia* post-therapy potentially impacted 12M-enriched LysoPS 20:4. Decreased *L. iners* post-therapy potentially influenced *Bifidobacterium* sp. levels at 12M. Hypoxanthine at 6M, which was depleted post-therapy, potentially affected *Peptoniphilus* sp. levels at 12M. Inosine, which decreased at 6M compared to Baseline, potentially influenced increased *S. agalactiae* at 12M. Notably, HPV-negative status at 6M potentially regulated 5-(12,15-Heneicosadienyl)−1,3-benzenediol abundance at 12M, which increased post-therapy.

We also identified potential time-lag effects among VMeta components (Fig. 5). For instance, ursodeoxycholic acid and HemiBMP 54:7; HemiBMP(16:2/16:2/22:3) levels at 12M were potentially regulated by 6M-enriched HemiBMP 54:7; HemiBMP(16:2/16:2/22:3); and HemiBMP 44:4; HemiBMP(14:0/14:1/16:3), respectively. Moreover, five 6M-depleted metabolites potentially impacted several metabolites at 12M, including interactions between guanosine 5′-diphosphate-D-mannose and 3,4-methylenedioxyamphetamine, inosine and TG 40:8; TG(12:3/12:3/16:2), ε-Caprolactam and LysoPE 17:1 to hypoxanthine, as well as docosatrienoic acid and adenosine monophosphate.

Furthermore, we identified 41 directional interactions that consistently existed across all three timepoints (Fig. 5). Specifically, HPV infection potentially impacted *Megasphaera* sp. and erucamide, both of which positively correlated with *L. crispatus* (Fig. 4; Table S2). Protoanemonin levels consistently interacted with *L. crispatus* abundance, with *L. crispatus* influencing protoanemonin levels. We also observed the impact of 5-(12,15-Heneicosadienyl)−1,3-benzenediol on *Megasphaera* sp. and LysoPS 16:0. *L. gasseri* and *F. vaginae* potentially regulated isoleucyl-leucine and LysoPS 20:4 levels, respectively. *L. iners* potentially modulated (Z,Z)−2-methyl-5-(8,11,14-pentadecatrienyl)−1,3-benzenediol, ursodeoxycholic acid, and inosine. We also identified potential *S. anginosus*→*U. parvum* and *S. agalactiae*→*Peptoniphilus* sp. interactions. Inferred regulatory networks among VMeta components included interactions following the directions: hypoxanthine→inosine, phosphoribosyl-AMP→maltotetraose→4-O-β-galactopyranosyl-D-mannopyranose, irbesartan→isorhynchophylline→docosatrienoic acid, and erucamide→oleoyl ethylamide→docosatrienoic acid.

## DISCUSSION

Our longitudinal study provides comprehensive insights into the dynamic changes of both VM and VMeta following therapeutic elimination of high-grade CIN. Moreover, there were no significant differences of VM and VMeta dynamics among those treatments, suggesting their similar impacts on vaginal microenvironments. As for specific variations post-therapy, we observed a gradual restoration of *L. crispatus*, accompanied by distinct metabolic shifts over the 12-month follow-up period. These findings reveal the temporal trajectory of vaginal microenvironment recovery and highlight potential mechanisms underlying the host-microbiome interactions during post-therapy rehabilitation.

The progressive increase in *L. crispatus*-dominated communities (CST_I) observed in our study aligns with previous reports indicating the restoration of vaginal *Lactobacillus* following CIN treatment (10–12). *L. crispatus* is widely recognized as the most protective *Lactobacillus* species against HPV infection and CIN progression (19–23), primarily due to its robust capacity to produce lactic acid, maintain low vaginal pH, and inhibit pathogenic microorganisms (24–26). Thus, the restoration of *L. crispatus* dominance represents a significant improvement in vaginal health following therapeutic intervention.

Interestingly, we observed increased abundance of several non-*Lactobacillus* species at 6 months post-therapy compared to the baseline, including *Bifidobacterium* sp., *S. anginosus*, *Megasphaera* sp., and *F. vaginae*. While some of these microorganisms have traditionally been associated with dysbiosis (3–7, 27, 28), recent studies emphasize the necessity to determine VM roles at strain level (29). Their transient increase might represent an intermediate stage in the restoration of vaginal homeostasis, potentially creating conditions favorable for subsequent *L. crispatus* colonization. This interpretation is supported by our dynamic Bayesian network analysis, which revealed potential regulatory relationships between these species and metabolites associated with vaginal health.

The concurrent reduction in organisms 6 months post-therapy frequently associated with vaginal dysbiosis (12, 30–32), such as *P. bivia*, *U. parvum*, *F. magna*, and *Peptoniphilus* sp., further confirms the trend toward restored vaginal health. *U. parvum*, in particular, showed a continuous decrease throughout the follow-up period. This is consistent with previous findings indicating decreased levels of those species post-therapy (12, 30). Thus, post-therapy dynamics of those species suggested sustained improvement in the vaginal microenvironment following therapies. The elimination of these potentially pathogenic microorganisms likely contributes to reduced inflammation and creates conditions conducive to *Lactobacillus* dominance.

The metabolomic changes we observed provide deeper insights into the functional implications of those microbial shifts. The early post-therapy period (Baseline to 6M) was characterized by the enrichment of several phospholipids (including HemiBMP 54:7, HemiBMP 44:4, and DGDG 34:1) and the depletion of some lipid metabolites (e.g., linoleic acid, ursodeoxycholic acid), purine metabolites (e.g., hypoxanthine, guanosine, inosine), and picolinic acid. This metabolic profile is distinct from that previously reported in HPV-positive women, who typically exhibited depleted phospholipid-related metabolites compared to HPV-negative controls (3, 13, 15). These findings suggest that therapeutic elimination begins to reverse these dysplasia-associated metabolic alterations as early as 6 months post-intervention.

The later phase of recovery (6M to 12M) revealed further metabolic refinement, with increases in flavonoids (naringenin chalcone), lysophospholipids (LysoPS 20:4, LysoPE 17:1), bioactive amides (erucamide, oleoyl ethylamide), and amino acids (several dipeptides). This is distinct from those identified in HPV-positive and CIN women, being featured by depleted amino acid metabolism (13–15). Some of these metabolites have been associated with anti-inflammatory and immunomodulatory properties in various biological contexts (33–35). Concurrently, we observed continued depletion of nucleotides (adenosine monophosphate, guanosine 5′-monophosphate), specific lipid metabolites (linoleic acid), and maltotetraose. Though the specific roles of those metabolites need further researches, recent studies provide some hints. For instance, maltotetraose facilitated the growth of *Gardnerella* sp. which is commonly associated with vaginal dysbiosis and higher risk of HPV infection (19, 20, 36). Collectively, these findings suggested progressive normalization of metabolic pathways disrupted during HPV infection and CIN development.

The strong correlations we identified between VM components and specific metabolites provide evidence for functional relationships within the vaginal ecosystem. *L. crispatus* abundance positively correlated with metabolites enriched at 12 months, including naringenin chalcone, various lysophospholipids, and several dipeptides. These correlations suggest that *L. crispatus* may either directly produce these metabolites or create conditions favorable for their production. Conversely, *L. crispatus* negatively correlated with nucleotide metabolites and certain lipids that decreased post-therapy, indicating potential antagonistic relationships. Particularly intriguing was the network of correlations between non-*Lactobacillus* species and specific metabolites. The positive correlations between *Megasphaera* sp., *Bifidobacterium* sp., *S. agalactiae*, *F. vaginae*, and specific lipid metabolites suggest complex interactions that may facilitate the transition toward *Lactobacillus* dominance. These findings challenge simplistic interpretations of vaginal health based solely on *Lactobacillus* abundance (29, 37). Furthermore, they highlight the importance of considering the entire microbial community and associated metabolic outputs when assessing vaginal health.

Our dynamic Bayesian network analysis revealed potential causal relationships between VM and VMeta components across timepoints. The finding that HPV-negative status at 6 months potentially regulated 5-(12,15-Heneicosadienyl)−1,3-benzenediol abundance at 12 months suggests that clearance of HPV infection has downstream effects on the vaginal metabolome that persist over time. Similarly, the influence of early post-therapy microbiota (such as *Bifidobacterium* sp.) on later metabolite levels indicates that the initial recovery phase sets the trajectory for subsequent metabolic restoration. The time-lag effects identified among metabolites themselves suggest the existence of metabolic cascades that unfold over months following therapeutic intervention. For instance, the impact of early changes in HemiBMP compounds on later metabolite levels indicates that the metabolic recovery process follows a programmed sequence rather than occurring simultaneously across all pathways. These findings emphasize the importance of longitudinal studies with multiple timepoints to fully capture the dynamics of recovery.

Consistent directional interactions identified across all three timepoints represent core regulatory relationships in the vaginal ecosystem. The finding that HPV infection potentially impacts *Megasphaera* sp. and erucamide levels, which, in turn, correlate with *L. crispatus* abundance, suggests a potential mechanism by which HPV influences vaginal health. Similarly, the consistent influence of *L. crispatus* on protoanemonin levels suggests that this metabolite may serve as a biomarker for *L. crispatus* activity in the vaginal environment.

Our study has several strengths, including its longitudinal design, comprehensive assessment of both microbiota and metabolome, and sophisticated analytical approach. By following women for 12 months post-therapy, we captured both early and late recovery phases, revealing distinct patterns of microbial and metabolic restoration. The integration of microbiome and metabolome data allowed us to explore functional relationships that would not be apparent from either data set alone. Furthermore, dynamic Bayesian network analysis provided insights into potential causal relationships that conventional correlation analysis cannot reveal.

However, several limitations should be acknowledged. First, while our sample size was sufficient to detect significant changes, larger cohorts would enable more robust subgroup analysis and potentially reveal additional patterns. In addition, there were no HPV-negative women as controls. Including healthy, HPV-negative women would have provided a reference point for “normal” VM and associated metabolome profiles, allowing for a more comprehensive interpretation of the recovery trajectory following CIN treatments. Specifically, such controls would help determine whether the post-therapy state at 12 months truly represents a return to a healthy vaginal microenvironment or merely an improvement from the dysbiotic state associated with HPV infection and CIN. The recruitment of appropriate controls is challenging in this context, as matching for age, sexual behavior, and other confounding variables is complex. Future studies should strive to include such control groups to strengthen the interpretation of restoration patterns observed post-therapy. Second, our study focused on women who successfully cleared HPV infection following therapy; the dynamics might differ in women with persistent HPV infection. Third, while our study utilized Dynamic Bayesian Network analysis to elucidate microbe-metabolite associations, we acknowledge analytical limitations in our approach. Alternative methods, including single-sample-based network statistics and tools incorporating microbial metabolic prior knowledge (such as MGCs, BGCs, GEMs, or functional prediction tools like PICRUSt2), were not implemented due to several constraints. The V4-V5 16S rRNA amplicon data have inherent limitations for species-level functional predictions, many reference databases inadequately represent strain-level vaginal microbiome diversity, and integrating metabolomic data with predicted functional capacity introduces additional uncertainty. These analytical constraints potentially limit the mechanistic interpretations of our observed microbe-metabolite relationships and represent an important consideration when evaluating our findings. Moreover, experimental validation would be necessary to confirm causal mechanisms. Future research should address these limitations and explore several promising directions. Studies incorporating host immunological markers would provide a more comprehensive understanding of the host-microbe-metabolite interactions following CIN therapy. Additionally, investigating the role of specific metabolites identified in our study (such as naringenin chalcone, erucamide, and various lysophospholipids) in modulating vaginal immunity and microbial compositions could reveal new therapeutic targets. Finally, comparative studies of different therapeutic approaches for CIN, including their impacts on VM and VMeta recovery, could inform clinical decision-making.

In conclusion, our study demonstrates that therapeutic elimination of high-grade CIN initiates a gradual restoration of vaginal health characterized by increased *L. crispatus* dominance and normalization of the vaginal metabolome. This recovery process follows a sequential pattern, with distinct early and late phases characterized by different microbial and metabolic shifts. The complex interactions between microbiota and metabolite dynamics highlight the importance of considering functional aspects of the vaginal microenvironment in assessing post-therapy recovery. These findings contribute to our understanding of host-microbiome interactions following therapeutic elimination of high-grade CIN. This further informs future strategies for monitoring treatment efficacy and promoting optimal recovery following CIN therapy.

## Data Availability

Raw data for VM analysis are available in the CNGB Sequence Archive (CNSA) under Project No. CNP0004255 and CNP0002763. The metabolomes datasets are available at CNSA (https://db.cngb.org/search/metabolize/METM0000175/)

## SUPPLEMENTAL MATERIAL

Supplemental Figures msystems.01190-25-s0001.pdf Fig. S1 to S4.

Table S1 msystems.01190-25-s0002.docx Contribution of treatment grouping to microbial and metabolic profiles at baseline.

Table S2 msystems.01190-25-s0003.csv Correlations between microbial components and associated metabolites.

ASM does not own the copyrights to Supplemental Material that may be linked to, or accessed through, an article. The authors have granted ASM a non-exclusive, world-wide license to publish the Supplemental Material files. Please contact the corresponding author directly for reuse.
