# Supplementary material for: One-year restoration of vaginal health: synergistic dynamics of microbiome and metabolome following the elimination of high-grade cervical intraepithelial neoplasia
Source: mSystems. 2025 Nov 17;10(12):e01190-25. doi: 10.1128/msystems.01190-25 (PMC12710359; doi:10.1128/msystems.01190-25)
Supplement: Supplemental Figures — Fig. S1 to S4. [file msystems.01190-25-s0001.pdf]

Fig S1. PERMANOVA for vaginal microbiota and metabolome. A-D. Four vectors of person-specific open taxonomic unit (OTU) and metabolite changes were calculated for each OTU and metabolite per individual as follows: $\log_2 (OTU_{6M}/OTU_{Baseline})$, $\log_2 (OTU_{12M}/OTU_{6M})$, $\log_2 (metabolite_{6M}/metabolite_{Baseline})$ and $\log_2 (metabolite_{12M}/metabolite_{6M})$. Then PERMANOVA was applied to analyze explained variance of several factors on these vectors.

Fig S2. Intra-individual dissimilarity between baseline and 6M as well as between 6M and 12M, according to microbial profiles.

Fig S3. Dynamics of 12 species at three timepoints. The point represents median value. Top and bottom error bars represent upper and lower quartile, respectively.

Fig S4. Intra-individual dissimilarity between baseline and 6M as well as between 6M and 12M according to metabolic profiles.

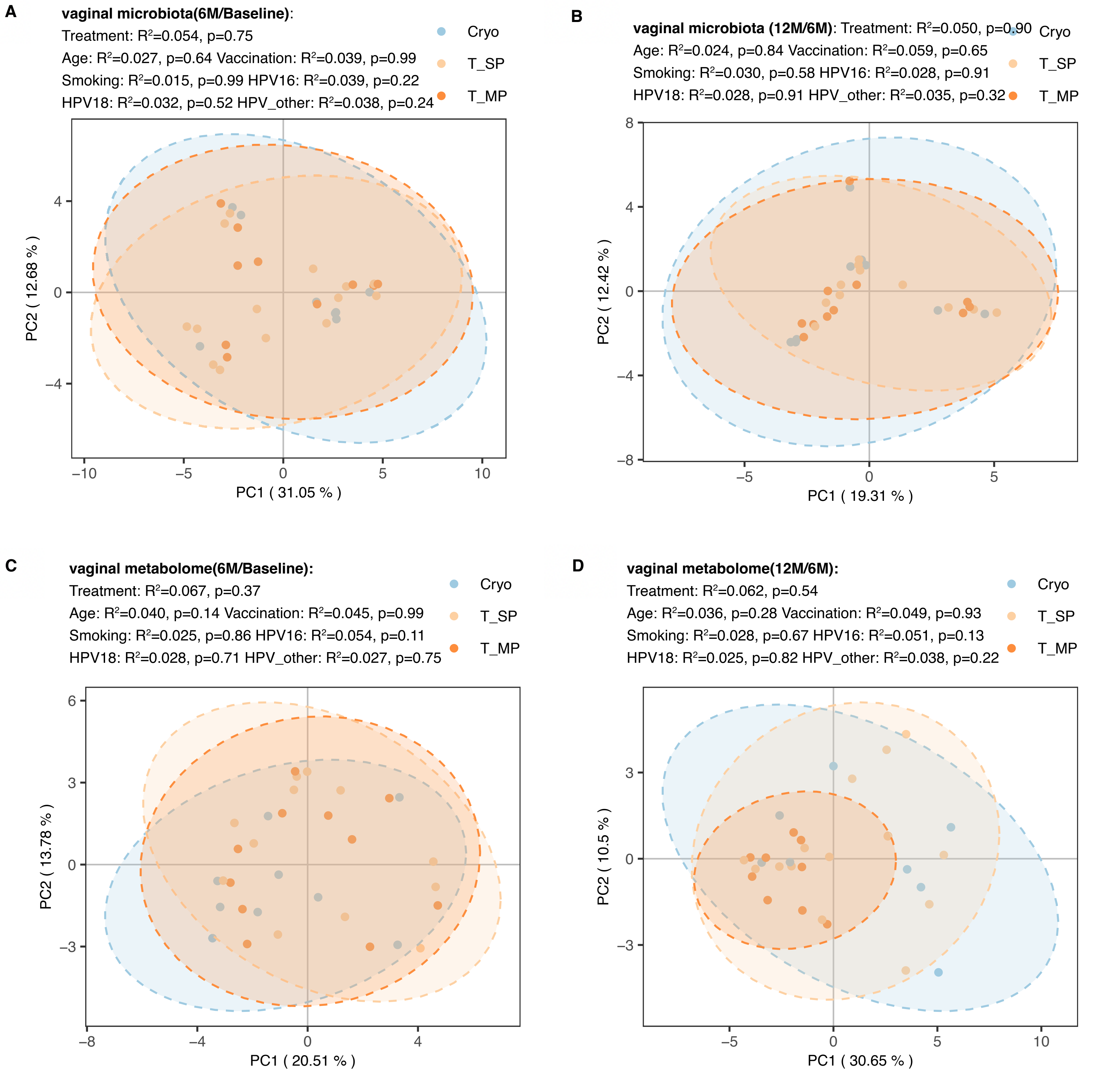
A
vaginal microbiota(6M/Baseline):
Treatment: R²=0.054, p=0.75
Age: R²=0.027, p=0.64 Vaccination: R²=0.039, p=0.99
Smoking: R²=0.015, p=0.99 HPV16: R²=0.039, p=0.22
HPV18: R²=0.032, p=0.52 HPV_other: R²=0.038, p=0.24
Cryo
T_SP
T_MP
PC2 (12.68 %)
PC1 (31.05 %)
B
vaginal microbiota (12M/6M): Treatment: R²=0.050, p=0.90
Age: R²=0.024, p=0.84 Vaccination: R²=0.059, p=0.65
Smoking: R²=0.030, p=0.58 HPV16: R²=0.028, p=0.91
HPV18: R²=0.028, p=0.91 HPV_other: R²=0.035, p=0.32
Cryo
T_SP
T_MP
PC2 (12.42 %)
PC1 (19.31 %)
C
vaginal metabolome(6M/Baseline):
Treatment: R²=0.067, p=0.37
Age: R²=0.040, p=0.14 Vaccination: R²=0.045, p=0.99
Smoking: R²=0.025, p=0.86 HPV16: R²=0.054, p=0.11
HPV18: R²=0.028, p=0.71 HPV_other: R²=0.027, p=0.75
Cryo
T_SP
T_MP
PC2 (13.78 %)
PC1 (20.51 %)
D
vaginal metabolome(12M/6M):
Treatment: R²=0.062, p=0.54
Age: R²=0.036, p=0.28 Vaccination: R²=0.049, p=0.93
Smoking: R²=0.028, p=0.67 HPV16: R²=0.051, p=0.13
HPV18: R²=0.025, p=0.82 HPV_other: R²=0.038, p=0.22
Cryo
T_SP
T_MP
PC2 (10.5 %)
PC1 (30.65 %)

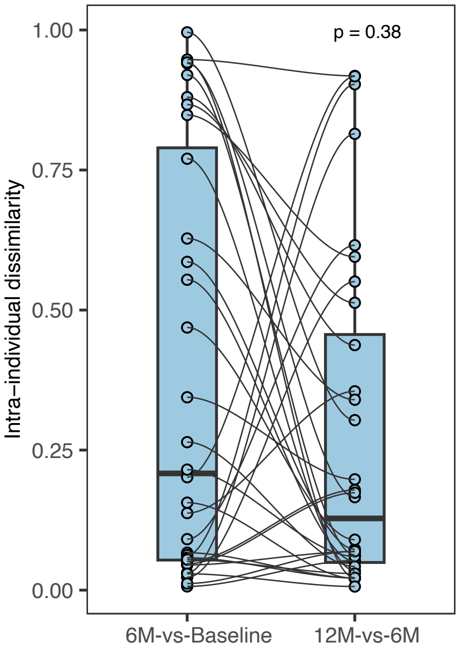
p = 0.38
Intra−individual dissimilarity
1.00
0.75
0.50
0.25
0.00
6M-vs-Baseline
12M-vs-6M

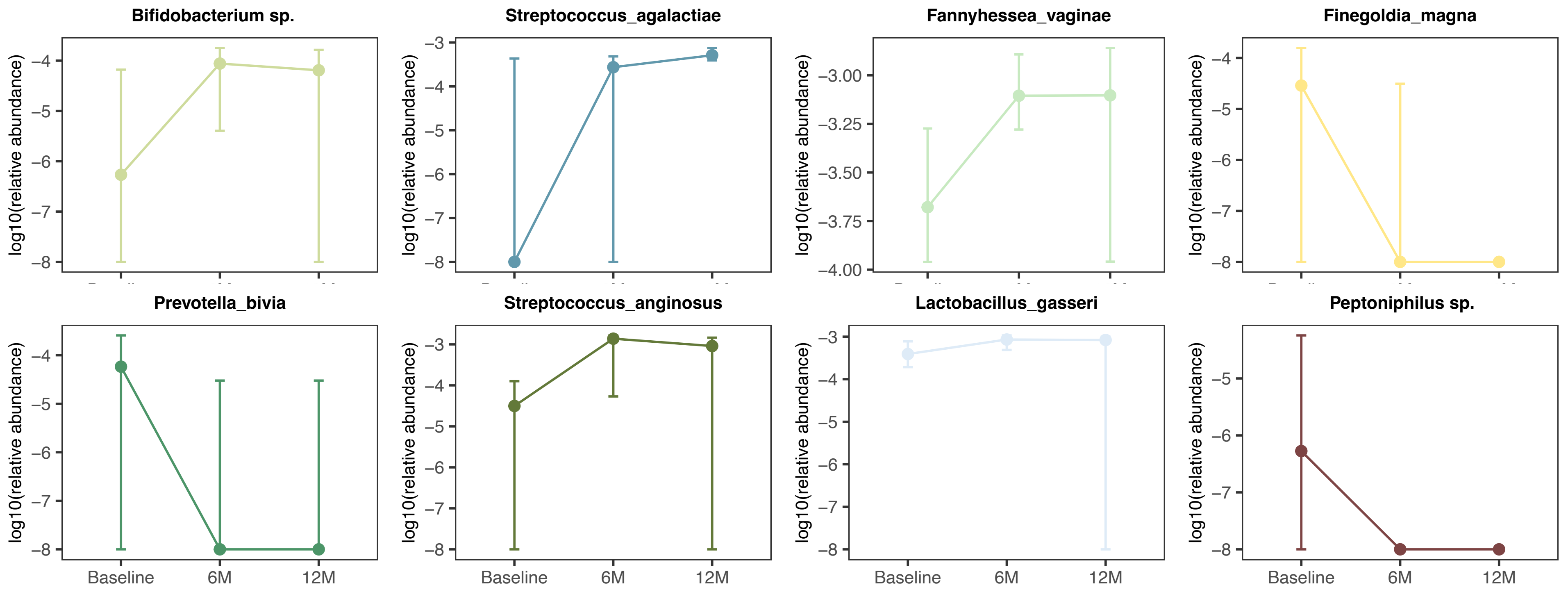

Bifidobacterium sp.
Streptococcus_agalactiae
Fannyhessea_vaginae
Finegoldia_magna
Prevotella_bivia
Streptococcus_anginosus
Lactobacillus_gasseri
Peptoniphilus sp.
log10(relative abundance)
Baseline
6M
12M

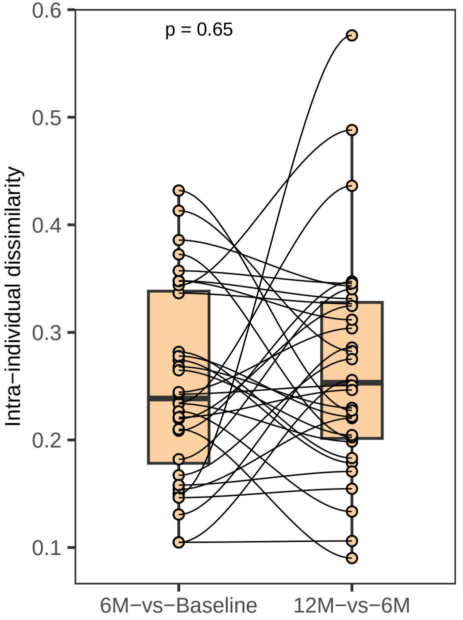

p = 0.65
Intra−individual dissimilarity
0.6
0.5
0.4
0.3
0.2
0.1
6M−vs−Baseline
12M−vs−6M
