# Supplementary material for: One-year restoration of vaginal health: synergistic dynamics of microbiome and metabolome following the elimination of high-grade cervical intraepithelial neoplasia
Source: mSystems. 2025 Nov 17;10(12):e01190-25. doi: 10.1128/msystems.01190-25 (PMC12710359; doi:10.1128/msystems.01190-25)
Supplement: Table S1 — Contribution of treatment grouping to microbial and metabolic profiles at baseline. [file msystems.01190-25-s0002.docx]

**Table S1. The contribution of treatment grouping to microbial and metabolic profiles at baseline**

|  | | **Cryo vs T_SP** | **T_SP vs T_MP** | **Cryo vs T_MP** |
| --- | --- | --- | --- | --- |
| **VM** | R^2^ | 0.017 | 0.010 | 0.002 |
|  | P | 0.81 | 0.93 | 0.99 |
| **Vaginal metabolome** | R^2^ | 0.027 | 0.019 | 0.042 |
|  | P | 0.79 | 0.85 | 0.53 |
